# Three-Dimensionally Printed Hydrogel Cardiac Patch for Infarct Regeneration Based on Natural Polysaccharides

**DOI:** 10.3390/polym15132824

**Published:** 2023-06-26

**Authors:** Jorge Loureiro, Sónia P. Miguel, Victor Galván-Chacón, David Patrocinio, José Blas Pagador, Francisco M. Sánchez-Margallo, Maximiano P. Ribeiro, Paula Coutinho

**Affiliations:** 1 CPIRN-IPG—Center of Potential and Innovation of Natural Resources, Polytechnic Institute of Guarda, 6300-559 Guarda, Portugal; jcloureiro97@gmail.com (J.L.); spmiguel@ipg.pt (S.P.M.); mribeiro@ipg.pt (M.P.R.); 2 CICS-UBI—Health Sciences Research Center, University of Beira Interior, 6201-001 Covilhã, Portugal; 3 Jesús Usón Minimally Invasive Surgery Center, 10071 Cáceres, Spain; vpgalvan@ccmijesususon.com (V.G.-C.); dpatrocinio@ccmijesususon.com (D.P.); jbpagador@ccmijesususon.com (J.B.P.); msanchez@ccmijesususon.com (F.M.S.-M.); 4 TERAV/ISCIII—Red Española de Terapias Avanzadas, 10071 Cáceres, Spain; 5 CIBER CV—Centro de Investigación Biomédica en Red-Enfermedades Cardiovasculares, 28029 Madrid, Spain

**Keywords:** konjac/gellan gum hydrogel, bioink, 3D printing, tissue engineering, myocardial infarction, cardiac tissue regeneration

## Abstract

Myocardial infarction is one of the more common cardiovascular diseases, and remains the leading cause of death, globally. Hydrogels (namely, those using natural polymers) provide a reliable tool for regenerative medicine and have become a promising option for cardiac tissue regeneration due to their hydrophilic character and their structural similarity to the extracellular matrix. Herein, a functional ink based on the natural polysaccharides Gellan gum and Konjac glucomannan has, for the first time, been applied in the production of a 3D printed hydrogel with therapeutic potential, with the goal of being locally implanted in the infarcted area of the heart. Overall, results revealed the excellent printability of the bioink for the development of a stable, porous, biocompatible, and bioactive 3D hydrogel, combining the specific advantages of Gellan gum and Konjac glucomannan with proper mechanical properties, which supports the simplification of the implantation process. In addition, the structure have positive effects on endothelial cells’ proliferation and migration that can promote the repair of injured cardiac tissue. The results presented will pave the way for simple, low-cost, and efficient cardiac tissue regeneration using a 3D printed hydrogel cardiac patch with potential for clinical application for myocardial infarction treatment in the near future.

## 1. Introduction

Cardiovascular diseases are the primary cause of death worldwide. Myocardial infarction (MI) provokes the obstruction of blood flow, leading to oxygen deficiency in the heart muscles and, hence, cell death [1,2]. In these situations, the limited regeneration ability of the adult cardiomyocytes impairs tissue regeneration, compromising heart functions [3].

In addition, both the pharmacological approaches and stem-cell-implantation-based therapy have drawbacks. For pharmacological approaches, it is well known that these do not restore normal heart function, working only as a means to reduce mortality. In relation to the stem-cell-implantation-based therapies, mesenchymal stem cells are used commonly for tissue regeneration, due to their immunomodulatory and vascular-repairing capabilities. However, the maintenance and the survival of transplanted stem cells is a significant challenge in regenerative medicine [4,5]. Several approaches have been explored in the production of new and alternative treatments able to restore normal heart function and reduce the morbidity associated with MI; such approaches have mainly been patches [4,6,7], injectable gels [8], or decellularized extracellular matrixes (ECM) [9]; however, the generation of engineering substitutes for highly vascularized cardiac tissues remains extremely challenging [10,11]. 

Engineered hydrogels are recognized due to the formation of 3D hydrophilic polymer networks that mimic the tissue microenvironments [12,13], which is the reason why they are extensively used in cardiovascular tissue engineering [8,14,15,16]. The selection of polymers is a critical step, considering the strict and specific requirements for supporting the mechanical stress of the cardiac tissue. Consequently, the use of a blend is recommended. The intermolecular interaction between polymers resulted in an increase in the hydrogels strengthening and toughening, thus obtaining enhanced scaffolds for regeneration of the damaged myocardium [17]. Natural or synthetic polymers, including collagen, gelatin, Matrigel, poly(2-hydroxyethyl methacrylate) (PHEMA), poly(N-isopropylacrylamide) (PNIPAAM) and polyethylene glycol (PEG), polyvinyl alcohol) (PVA), polyvinyl chloride (PVC), polycaprolactone (PCL), and polylactic acid (PLA), have been widely reported as being good options for the production of the hydrogels [18,19,20]. 

Such hydrogels have been produced through conventional methodologies, namely gelation, solvent casting/particulate leaching, freeze-drying, and gas foaming. However, such approaches are unable to create load-bearing structures that can mimic the complex hierarchical architecture and mechanical properties of the native extracellular matrix [21,22]. Thus, 3D printing techniques have emerged in recent years, being an advanced fabrication technique for producing hierarchically ordered structures using hydrogels for tissue engineering purposes. Such techniques have allowed precise control over the design and final structure of the scaffolds. They enable the creation of structures that can replicate the ECM’s complex architecture and physical properties [21,22,23]. Some pioneering works have widely reported the use of printable biomaterials, as well as 3D printing approaches for the fabrication of functional living constructs with 3D customized architecture [24,25]. In this field, the selection of the bioinks compositions is considered a critical step in the printing process. Hence, the selected biomaterials should be printable, with both high structural integrity and reproducibility, and simultaneously should mimic the extracellular matrix of the human heart tissue, promoting cell proliferation and differentiation [26].

In this study, a 3D printed hydrogel was produced using the extrusion printing process. Herein, we formulated a functional bioink based on the natural polysaccharides gellan gum (GG) and konjac glucomannan (KGM), due to their biocompatibility, structural integrity, and friendly crosslinking [26,27]. Human umbilical vein endothelial cells (HUVECs) were used, due to their angiogenic character, as well as their other crucial biological functions [28]. In addition to this, HUVECs also have the ability to transdifferentiate into cardiac muscle [29,30], as well as fuse with surrounding noncardiomyocytes, making those cells re-enter the cell cycle [31]; this makes them suitable candidates for being integrated in cardiac patches. It is worth noting that this natural polysaccharide combination had not yet been explored in the production of 3D printed structures for cardiac tissue regeneration purposes, and our preliminary results [32] highlighted their potential to be used as a simple, low-cost bioink for 3D printing tissue-engineering scaffolds. The therapeutic potential of the 3D printed hydrogel to be used in cardiac tissue regeneration was characterized according to their physicochemical and biological properties, namely regarding their mechanical performance and the suitability for propelling the HUVECs’ cell adhesion and proliferation.

## 2. Materials and Methods

### 2.1. Materials

Absolute ethanol (EtOH), hydrochloric acid of ≥37% purity, potassium chloride, potassium phosphate dibasic trihydrate, and sodium chloride were obtained from Laborspirit, Loures, Portugal. Bovine serum albumin of ≥98% purity, as well as fetal bovine serum, were obtained from Biowest, Nuaillé, France. Crosslinking Agent (50 M CaCl_2_) was obtained from Cellink, Gothenburg, Sweden. Dimethyl Sulfoxide (DMSO) of ≥99% purity, as well as disodium hydrogen phosphate, were obtained from VWR chemicals, Radnor, PA, USA. Dulbecco’s modified Eagle’s medium/nutrient mixture F-12 ham (DMEM-F12), 3-[4,5 Dimethylthiazol-2-yl]-2,5-diphenyl tetrazolium bromide (MTT), paraformaldehyde ≥36% (*w*/*v*), tris(hydroxymethyl) aminomethane and trypsin powder (porcine) 1:250 were purchased from Sigma-Aldrich, St. Louis, MO, USA. Endothelial cell basal medium was obtained from PromoCell. GG was obtained from Alfa Aesar, Haverhill, MA, USA. Hoechst 33342^®®^ was purchased from Invitrogen (Carlsbad, CA, USA). Glutaraldehyde 25 % (*w*/*v*) and sodium sulfate were obtained from Acofarma, Madrid, Spain. HUVECs and normal human dermal fibroblasts (NHDF) were obtained from Promo Cell Labclinics, S.A, Barcelona, Spain. KGM was obtained from Sports Supplemented Ltd., Colchester, UK. Magnesium chloride hexahydrate and sodium bicarbonate were obtained from Panreac, Barcelona, Spain. Penicillin-streptomycin-amphotericin B was obtained from Lonza Walkersville, MD, USA. Potassium dihydrogen phosphate was obtained from VWR, Radnor, PA, USA.

### 2.2. Production of 3D Printed Hydrogel Cardiac Patch

#### 2.2.1. Production of the Hydrogel Cardiac Patch Using the Conventional Gelation Method

To produce the scaffold using the conventional method of gelation, solutions of GG and KGM were prepared and blended. Firstly, the optimization of GG concentration was performed and then KGM was added. Different concentrations of 1–5% (*w*/*v*) GG were tested, aiming to evaluate its printability. After this optimization process, the selected concentrations were 3% of GG and 1% of KGM, whereupon the polysaccharides were dissolved in aqueous solutions at 90 °C, following an autoclave process of 120 °C for 30 min. Then, 1 mL of solution was taken into a cast to produce the patch. After gelation, the hydrogel was frozen at −20 °C and lyophilized overnight to create the conventional scaffold (CS).

#### 2.2.2. Production of the Hydrogel Cardiac Patch Using a 3D Printing Method

The 3D printing method used a BioX bioprinter (Cellink, Gothenburg, Sweden) and followed the same procedure as described in Section 2.2.1 to prepare the GG/KGM solution. After this, the solution was transferred to a syringe that had been preheated at 70 °C. The syringe with GG/KGM solution was then transferred to the holder. The 3D model (Supplementary Figure S1) was a square-based prism measuring 20 × 20 × 0.4 mm, with a 48% rectilinear infill. The printer was set to print with a 0.41 mm nozzle and a 0.2 mm layer height, at an extrusion temperature of 65 °C (which was over the gelation temperature) and room temperature of the bed, at a pressure of 80 kPa and with a 2.5 mm/s printing speed. After printing, the samples were immersed in a CaCl_2_ solution for 5 min and then washed and stored in distilled water. Later, the hydrogel was frozen at −20 °C and lyophilized overnight to produce the 3D printed scaffold (BS).

### 2.3. Physical and Morphological Characterization

#### 2.3.1. Attenuated Total Reflectance–Fourier-Transform Infrared Spectroscopy Analysis

The chemical characterization of both the polymers and the freeze-dried samples were carried out using Attenuated Total Reflectance–Fourier-Transform Infrared Spectroscopy (ATR-FTIR) with a Perkim Elmer ATR-FTIR Spectrum-Two. The ATR-FTIR chemical characterization was undertaken using the determination of the main spectral peaks’ displacement.

#### 2.3.2. Surface Morphology Characterization

The scaffolds morphology was characterized using scanning electron microscopy (SEM). The samples were freeze-dried (−80 °C) in a vacuum for 3 h. After that, samples were mounted onto aluminum stubs and analyzed using a scanning electron microscope (SEM, Tabletop microscope TM 3030Plus Hitachi, Toyo, Japan) at various magnifications. 

#### 2.3.3. Determination of Mechanical Properties

The mechanical properties were determined using a Texture Analyzer (TA-XT Plus, Stable Micro Systems, Surrey, UK). The assays were performed following the protocol described by Kianfar, Ayensu and Boateng (2014) [33], with some adaptations. In short, a 1″ cylindrical probe was used to compress samples (i.e., the lyophilized and rehydrated cardiac scaffolds) using the following settings: pretest speed (0.1 mm/s); test speed (0.1 mm/s); post-test speed (1 mm/s); strain (50%); trigger force (0.01 N); and mode (return to start). The adhesiveness (negative area) was determined from the resultant force–time plot in accordance with the procedure described by Hurler et al. (2012) [34]; in addition, the Young’s Modulus (E) was determined using the stress–strain relation, in accordance with the procedure already described in the literature [35]. The measurements were performed in triplicate, and at room temperature. In these assays, the BS had a cubic shape, with a size of 1 × 1 × 1 cm, using the same printing settings (with the exception of the printing speed, which was 6 mm/s).

#### 2.3.4. Swelling and Biodegradation Profile

The swelling ratio was characterized using a previously described method, with slight modifications [36]. In short, a known weight (W0) of each hydrogel was immersed in 5 mL of the updated simulated body fluid (USBF) solution [37] at 37 °C (Edmund Bühler GmbH TH 15 incubator hood, Bodelshausen, Germany). USBF is a revised version of simulated body fluid, which had been submitted to the Technical Committee ISO/TC150 of the International Organization for Standardization, with detailed instructions for its preparation. At predetermined intervals, the swollen samples were removed from the solution, and excess fluid was removed from the surface, and weighed again (Wt). The swelling was evaluated using the following equation:

Swelling ratio (%) = ((Wt − W0)/W0) × 100(1)

Additionally, the biodegradation of scaffolds was determined. W0 of each scaffold was immersed in 5 mL USBF at pH = 7.4 and at 37 °C. At predetermined intervals (1, 3, 7, 14, and 30 days), the samples were taken off the USBF solution, lyophilized, and then weighed (W_f_) again [38]. Biodegradation was finally determined using the following equation:

Biodegradation (%) = [(W_f_ − W_i_)/W_i_] × 100 (2)

### 2.4. Characterization of the Biological Profile of Scaffolds

#### 2.4.1. Characterization of the Cytotoxic Profile of Scaffolds

The cytotoxic profile of both the 3D printed and conventionally produced scaffolds were characterized using the MTT assay, which was performed in accordance with the guidelines set by the International Organization for Standardization (ISO) 10993-5 standard. A small piece of the hydrogel measuring 2 × 2 mm was placed in each well of a sterile 96-well plate. NHDF cells and HUVECs were seeded at a density of 2 × 10^4^ cells/well, and the plates were incubated at 37 °C under a 5% CO_2_ humified atmosphere for 24 h. After the 24 h incubation, the medium was removed, and 50 µL of MTT (5 mg/mL in PBS) was added to each sample, followed by incubation for 4 h in the same conditions previously described. Finally, MTT was removed, and cells were treated with 200 µL of DMSO (0.04 N) for 30 min. Then, the absorbance at 570 nm was measured using a microplate reader (Biorad xMark microplate spectrophotometer, Waltham, MA, USA). As a negative control, nontreated cells (K^−^) were used (i.e., cells with culture medium), and the positive control (K^+^) used EtOH to induce cell death.

#### 2.4.2. HUVECs Internalization within 3D Printed Scaffolds

Confocal laser scanning microscopy (CLSM) was used to visualize the cell distribution within hydrogels. HUVEC Cells (1.6 × 10^4^ cells/well) were seeded in hydrogels in μ-Slide 8-well Ibidi imaging plates (Ibidi GmbH, Planegg/Martinsried, Germany) on the surface of the hydrogel. After 48 h, the nucleus of the cells were stained with Hoechst 33342 (2 μM, Thermo Fisher Scientific, Waltham, MA, USA). Then, the imaging experiments were performed, using a Zeiss LSM710 laser scanning confocal microscope (Carl Zeiss AG, Oberkochen, Germany), whereupon consecutive z-stacks were acquired. The 3D reconstruction and image analysis were performed using Zeiss Zen 2010 software.

#### 2.4.3. Scratch Assay

HUVECs migration was evaluated through the in vitro scratch assay, validated for the analysis of cell migration [39]. This was performed as described by Chen, et al. [40], where 2.5 × 10^5^ cells/well were seeded in a 24-well plate with 1 mL of culture medium until confluence was attained. Then, a linear scratch was generated in the monolayer with a sterile 20 µL pipette tip. Any cellular debris were removed by washing the plate with PBS. Then, 1 mL of fresh medium was added to the cultures, and a scaffold sample (0.5 cm × 0.5 cm) was placed in contact with cells, which were then incubated at 37 °C inside an incubator with a 5% CO_2_ humidified atmosphere for 24 h. Cell migration was determined after 0, 3, 6, 18, and 24 h using an Optika inverted light microscope equipped with an Optikam B5 digital camera (Bergamo, Italy). Wound closure rate (initial wound area—wound area at 24 h)/initial wound area) was analyzed and calculated using ImageJ (Scion Corp., Frederick, MD, USA) and presented as a relative migration compared with t = 0, which was considered as 100%.

### 2.5. Statistical Analysis

Each experiment was performed in triplicate (*n* = 3). A two-way ANOVA, followed by a Bonferroni and Tukey post hoc test (*p* < 0.05), was used for cytotoxicity and stability assays, respectively. Data were considered significant at *p* < 0.05 (*). The data and statistical analysis were obtained using GraphPad Prism 9.2 for Windows (Graphpad Software, San Diego, CA, USA).

## 3. Results and Discussion

### 3.1. Scaffold Fabrication

**Figure 1:**
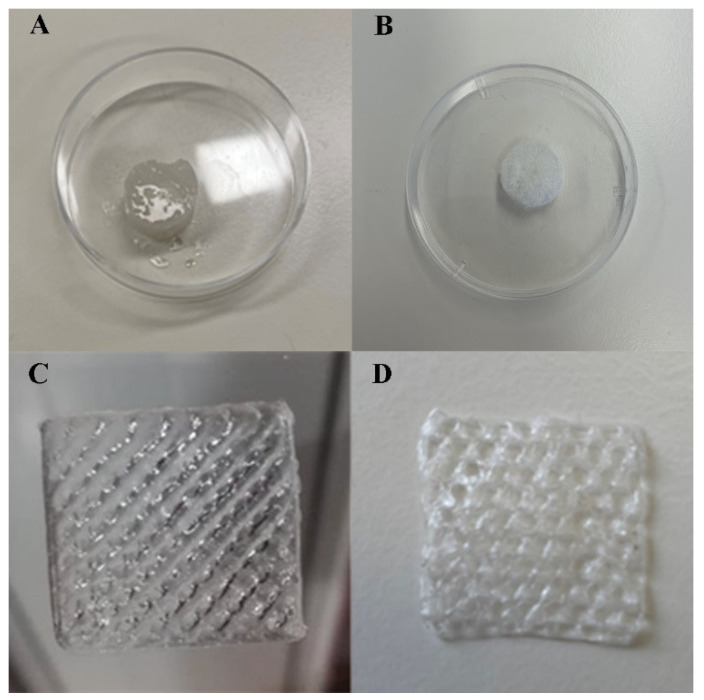
Results for scaffold fabrication: (**A**) Conventional Scaffold (CS) prior to lyophilization; (**B**) CS lyophilized; (**C**) 3D printed Scaffold (BS) prior to lyophilization; (**D**) BS lyophilized.

Through the macroscopic images of the produced scaffold in Figure 1, it was possible to observe that the conventional method resulted in more moisturized and opaque scaffolds than those that had been 3D printed prior to lyophilization. This was related to the immersion in calcium solution, resulting in an improvement of the resolution and 3D polymeric network stability by means of ionic interaction with the cations. Furthermore, it was also noticed that the lyophilization process did not compromise the scaffold shape. In fact, the freeze-drying process has typically been used to create porous architecture scaffolds, preserving their structure [41]. In addition, as expected, the 3D printed method demonstrated an ability to produce scaffolds with a controlled and reproducible shape, with a pore size within the range of 200 µm. Indeed, the natural polysaccharide-based bioink, the extrusion printing, and printing settings each allowed the spatial control on the deposition of biomaterials, promoting the fabrication of a functional scaffold with 3D customized architecture. Such results supported the suitability of the polysaccharides as components of bioinks, as well as their sufficient extrudability to achieve the required physical and mechanical stability of the 3D printed scaffolds [42].

### 3.2. Physical and Morphological Characterization

#### 3.2.1. ATR-FTIR Analysis

In the IR spectrum of KG, the more representative peaks appeared at 3341 and 2893 cm^−1^ displacement, corresponding to the stretching vibration of hydroxy (–OH) and methyl and methylene (–CH_3_ and –CH_2_), respectively [43,44,45]. In addition, it presented a peak at 1735 cm^−1^, which was a consequence of the asymmetric stretching vibration of carbonyl groups in acetyl. Another peak, corresponding to carbon–oxygen (C–O–C) stretching vibration in the six-member ring, was observed at 1634 cm^−1^. Additionally, two peaks were present at 1412 cm^−1^, corresponding to (C–H) bending of methylene (–CH_2_) carbons, and at 1371 cm^−1^, corresponding to methyl (–CH_3_) out-of-plane symmetric bending deformation, or umbrella peak. In addition, there was a peak at 1243 cm^−1^ which corresponded to the stretching vibration of C–O acetylene group, and other peaks at 1152 and 1021 cm^−1^, that were caused by the C–O bond. The characteristic absorption bands of the mannose in the konjac appeared at 871 and 804 cm^−1^.

From the IR spectrum of GG, two peaks were present at 3332 and 2883 cm^−1^, corresponding to the stretching vibration of hydroxyl (–OH) and methyl and methylene (–CH_3_ and –CH_2_), respectively [46,47]. Furthermore, two peaks were produced at 1605 and 1404 cm^−1^ by the symmetric and asymmetric stretching vibration of carbonyl groups in carboxylic acid salt. Several peaks were also caused at 1298, 1149, and 1019 cm^−1^, with the stretching vibration of the C–O bond.

In the IR spectrum of the GG/KGM hydrogel (Supplementary Figure S2), there were several peaks present at 3333, and 2922 with 2880 cm^−1^, corresponding to the stretching vibration of hydroxy (–OH) and methyl and methylene (–CH_3_ and –CH_2_), respectively [48,49,50]. A peak was present at 1725 cm^−1^ as a consequence of the asymmetric stretching vibration that corresponded to the carbonyl groups of the acetyl group. This peak was accompanied by two other peaks at 1605 and 1406 cm^−1^, which were produced by the symmetric and asymmetric stretching vibration of carbonyl groups in carboxylic acid salt. Additionally, another peak at 1378 cm^−1^ corresponded to methyl (–CH_3_) out-of-plane symmetric bending deformation, or umbrella peak. Three peaks caused by the stretching vibration of C–O of acetyl groups appeared at 1303, 1135, and 1019 cm^−1^, where the last of these corresponded to the CH_2_–OH bond. The peak at 1243 cm^−1^ was assigned to the asymmetric stretching of the acetyl chemical group. The characteristic absorption bands of the mannose in the konjac appeared at 879 and 799 cm^−1^. According to these results, a molecular restructuration could be present in this hydrogel, but must be confirmed with additional experiments.

#### 3.2.2. Surface Morphology Characterization

**Figure 2:**
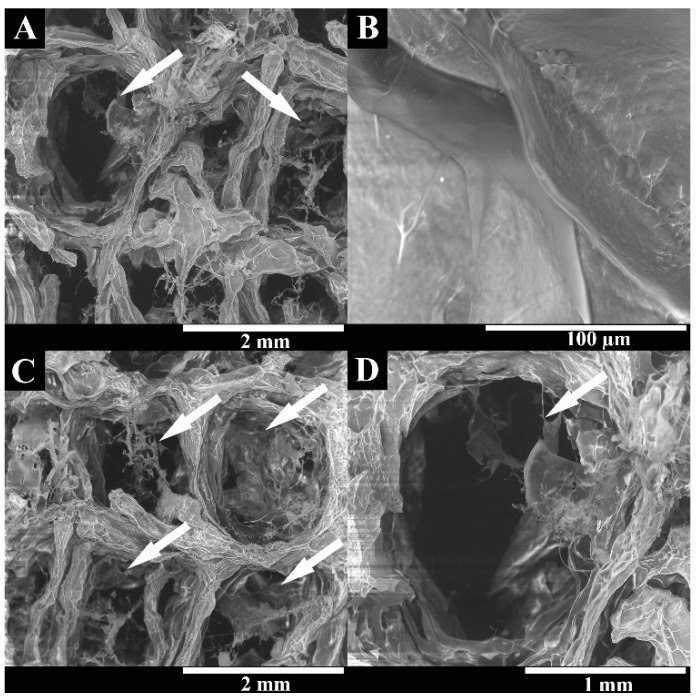
Representative SEM images of the lyophilized BS at different magnifications, showing the surface roughness and interconnected polymeric network. White arrows indicate the scaffolds pores. (**A**–**D**) represent the superficial porous structure of BS at different magnifications (**A**—40×; **B**—80×; **C**—40× and **D**—100×).

It has been widely reported in the literature that biomaterial surface topography is a crucial parameter influencing cell adhesion and proliferation [51,52]. In this study, the surface of 3D printed scaffolds (BS) was characterized through SEM analysis. The printing process demonstrated suitability for the production of 3D hydrogels based on natural polysaccharides, once presented with a rough surface with an interconnected porous network (Figure 2). The 3D scaffold showed roughness and irregularities in its surface, suggesting it possessed a high number of anchorage points suitable for protein adsorption, cell anchorage, and the production of extracellular matrix compounds [53,54,55]. 

#### 3.2.3. Determination of Mechanical Properties

**Figure 3:**
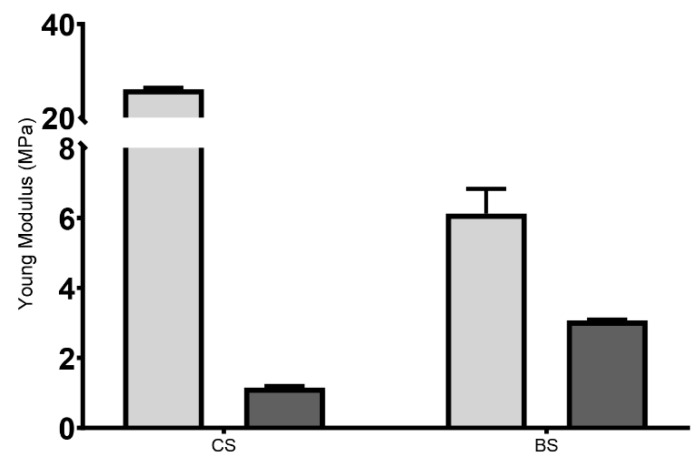
Young’s modulus of the 3D printed hydrogel. Light grey represents the lyophilized scaffolds, and dark grey represents the rehydrated ones.

The cardiac patches for tissue regeneration are subjected to considerable mechanical load and must be flexible, elastic, and mechanically stable [56]. The E module results have been presented in Figure 3. For lyophilized scaffolds, the results indicated that the E module of CS (26.077 ± 0.596 MPa) was about four times higher than the BS (6.121 ± 1.228 MPa). Ionic crosslinking may be responsible for this higher firmness, but the mesh (i.e., the infill pattern of the BS samples) will strongly affect this behavior, too. Nevertheless, when the scaffolds were rehydrated, the E values were significantly lower, with 3.069 ± 0.056 MPa and 1.154 ± 0.082 MPa for BS and CS, respectively. This suggested that, once rehydrated, the produced hydrogel was able to recover its flexibility. In addition, it reinforced the ability of 3D printing process to allow a controlled deposition of the bioink when producing the scaffold, resulting in a more homogeneous and interconnected polymeric network than the conventional method, and consequently resulting in a lower difference between dry and hydrated state. Such evidence indicated that the produced 3D printed hydrogel would be suitable for implantation [57]. 

Such low E values obtained for the produced hydrogels were in agreement with those available in the literature, where the E module of native human heart ranges are indicated as ranging from 0.02 to 0.5 MPa [58]; as such, an E value between 7 and 20 MPa is suitable for cardiac tissue engineering applications [56,59,60,61,62,63]. In addition, another study reported hydrated scaffolds with E values ranging from 1 to 5 MPa [64]. 

In general, the results obtained for E module indicated that the application of the 3D printed hydrogel was not compromised, as the scaffolds did not exhibit brittleness [33]. In addition, it was expected that the biodegradability of the polymer occurred simultaneously and synchronously with cell migration and proliferation, thus changing the mechanical characteristics of the scaffolds along with cellular activity and proliferation.

Regarding adhesiveness, lyophilized scaffolds had none, as expected. The rehydrated BS showed adhesiveness values of (−0.515 ± 1.121) N·s. Such parameters could assess the retention time of hydrogel at the implantation site. It has been previously described that the polymer concentration is closely related to its adhesive characteristics, wherein scaffolds with increasing polymer concentrations have better adhesive behavior [65,66]. In addition, the adhesiveness of BS has been of great interest because the adhesive materials are more likely to promote cell adhesion and avoid scar tissue formation, which is essential in cardiac tissue regeneration, and supports the successful implantation of a 3D printed scaffold with suitable mechanical properties for cardiac tissue regeneration. These results (when combined with the E values) indicated that these 3D printed cardiac hydrogel could be safely handled and applied without losing their structure, as well as provide the mechanical support for the weakened ventricular wall [67].

#### 3.2.4. Swelling and Biodegradation

The swelling profile of a biomaterial is the property that gives information about the polymeric matrix’s behavior when placed in contact with body fluids [68]. The ability of hydrogels to absorb water is widely recognized in the literature, since hydrogels are defined as a class of water-swollen polymers with high water content and physical properties similar to soft tissue [69]. In addition, the equilibrium swelling capacity echoes its ability to transport water, nutrients, and metabolic wastes between the cells and the medium [70]. 

**Figure 4:**
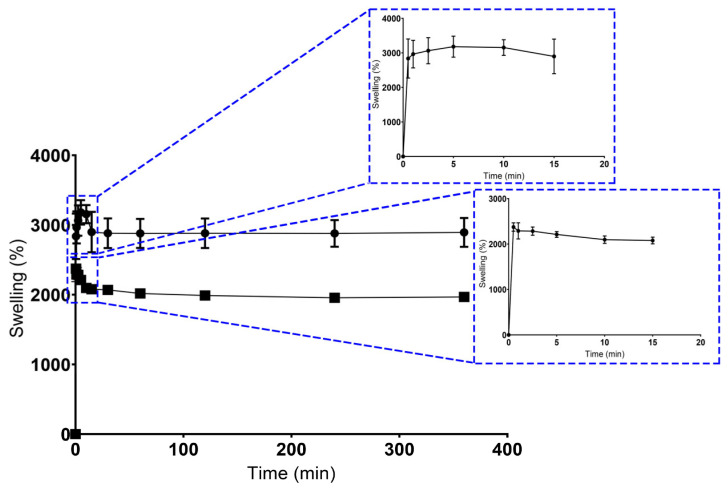
Results for swelling ratio. Dots represent CS’ data, and squares represent the BS’ data.

As a consequence, the swelling profile of the scaffolds produced through both techniques were characterized in contact with USBF, as shown in Figure 4. Concerning the maximum water absorption ability, the CS presented higher percentage values (3180.33 ± 304.03)% in comparison to the BS scaffold (2374.67 ± 92.36)%. After this maximum peak, both scaffold formulations exhibited a sustained swelling profile for at least 360 min. 

In general, the higher water content in scaffolds reflects its high hydrophilic character, as well as the increased presence of the OH groups on the polymeric networks of GG and KGM, as has been reported elsewhere [47,71,72].

Such differences between CS and BS scaffolds can be explained essentially by the precise control over structural properties, geometry, microarchitecture, pore size, and pore interconnectivity, and verified using the 3D printing methodology [73]. Moreover, the controlled swelling profile of BS may be related to a more homogeneous distribution of polysaccharides across the hydrogel structure and the ionic crosslinking method used, which implies the formation of ionic bonds responsible for increasing its cross-linking density, as well as reducing its swelling capacity [46]. 

The production of structures with defined architecture, pore size, and interconnectivity was only achieved in BS scaffolds. Unlike the conventional method, the BS scaffolds promoted the production of porous matrices with unstable physical and mechanical properties, which was in accordance with the swelling ability. In addition, higher swelling percentage values were considered undesirable for tissue engineering purposes, because an uncontrolled swelling profile can compromise the structural stability of scaffolds, which will affect the tissue formation process [74]. 

It is also important to note that these findings agree with the porosity results that have been defined in previous studies [52], where a direct influence of the production method on the scaffolds’ porosity was verified.

**Figure 5:**
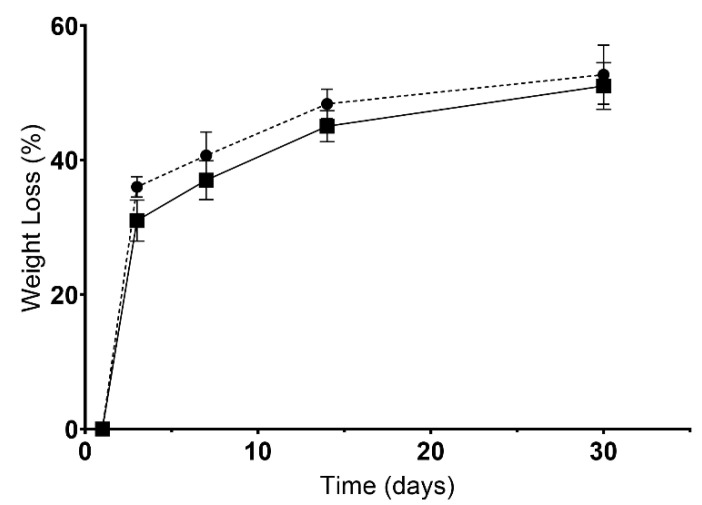
Results for biodegradation (weight loss). Dots represent CS’ data, and squares represent the BS’ data.

On the other hand, the biodegradation profile of scaffolds was also characterized, since a given biomaterial must be biodegradable at a controlled rate without inducing adverse cytotoxic effects. As such, in Figure 5, it is possible to notice that no statistical differences were observed between the CS and BS scaffolds. After 30 days of being immersed in USBF, CS presented a weight loss of 52.7 ± 4.4%, whereas BS lost 51.0 ± 3.5%. The results seem to indicate a controlled degradation of the polymers inside the scaffold, which is essential for cell incorporation into this scaffold. In addition, a controlled degradation profile is desirable because the biomaterial must guarantee the provisional support for cell anchorage, proliferation, and differentiation [75].

Overall, data obtained for mechanical characterization when integrated with the swelling and biodegradation profile revealed that this 3D printing technique allowed for the production of hydrogels with a typical performance sufficient to be confirmed for natural polymeric hydrogels, regarding the plasticity, the initial burst for fluid absorption, and stability in physiological conditions, as well as to act as a provisional structural matrix for promoting cardiac tissue regeneration. 

### 3.3. Characterization of the Biological Profile of Scaffolds

#### 3.3.1. Characterization of the Cytotoxic Profile of Scaffolds

**Figure 6:**
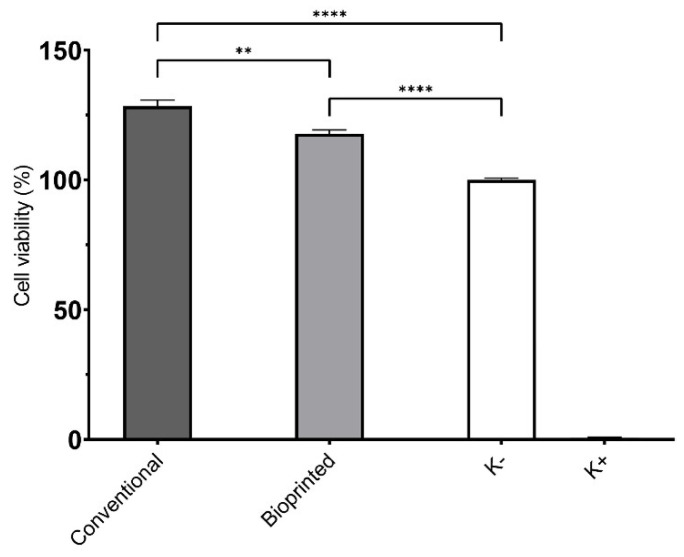
Results for HUVECs cell viability using MTT assay. Data are presented as mean ± standard deviation, *n* = 5, ** *p* < 0.01 and **** *p* < 0.0001.

The biocompatibility of scaffolds is fundamental for their application in the biomedical field. In this study, cell cytocompatibility was characterized by seeding NHDF and HUVEC cells in contact with the scaffolds. NHDFs were used in the first screening of the materials, in accordance with ISO 10933-5, whereas HUVECs were used due to their pivotal role in cardiac tissue formation. The results outlined that both scaffolds produced were biocompatible for NHDFs (data not shown) and HUVECs cells (Figure 6). In contact with NHDF, the cell viability percentage values were 135.3 ± 4.7% for CS, and 128.8 ± 3.3% for BS, whereas for HUVECs cells the values of 128.4 ± 2.4% for CS, and 117.8 ± 1.5% for BS were obtained, when compared to control group (i.e., untreated cells).

It is evident that the production method neither influenced the biological properties of the scaffolds nor reinforced the biocompatible character of GG and KGM, as has been reported elsewhere [27,47,76]. Moreover, GG and KGM are well described for their bioactivity, especially for enhancing cell proliferation, and other authors have already used GG for drug delivery and tissue engineering [26,47,55,71]; meanwhile, KGM has been used in wound healing purposes [27] and as a scaffold for vascularized bone tissues [77].

#### 3.3.2. Characterization of HUVECs Adhesion and Internalization

**Figure 7:**
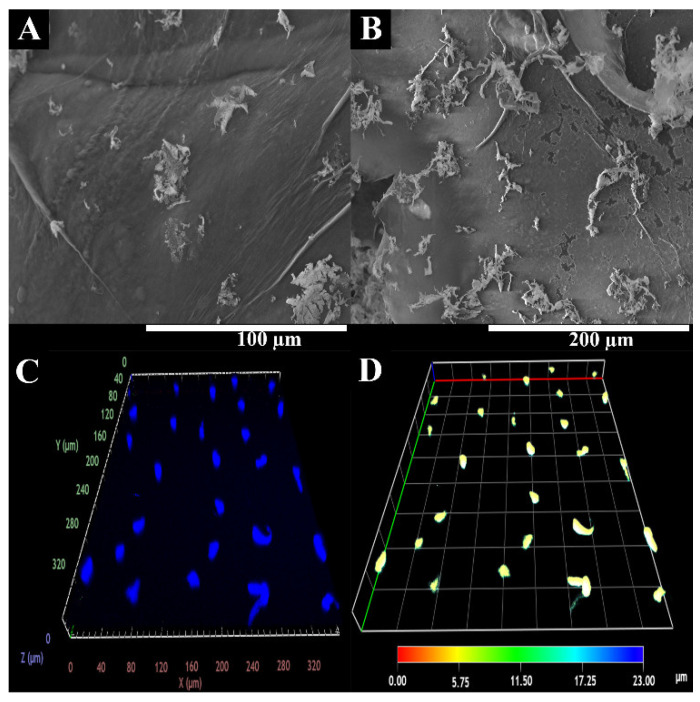
Representative SEM image of HUVECs adhesion at BS surface. (**A**,**B**) at different magnifications; CLSM images of HUVECs internalization into BS. (**C**,**D**) at different depth.

Considering the results we obtained, cell interaction with the BS surface was evaluated through SEM analysis. Figure 7 shows that the hydrophilic character of natural polysaccharide-based hydrogel and surface roughness of the hydrogel was suitable for cell adhesion and proliferation. A cardiac hydrogel must ensure cellular attachment, growth, and migration by acting as a 3D scaffold matrix. Apart from this, the biocompatible profile was also rather important [5]. In fact, after 48 h, it can be observed that the cells adhered to the material’s surface, establishing interconnections between them that contributed to the formation of endothelium, which regulates the mass transport exchange as well as controls the blood flow; this is fundamental for the in vitro fabrication of vascularized cardiac tissues [52,78].

The literature suggested that the environmental cue and topographic properties control the endothelial cell behavior, highlighting that endothelial cells are very prone to topological responses [79,80,81].

In addition, HUVECs migration and proliferation within BS were also characterized using confocal laser scanning microscopy (CLSM) analysis. The CLSM images (Figure 7) show that cells were able to migrate into the BS structure. Further, the depth-color-coding images also confirmed that the HUVECs cells migrated within the hydrogels. Most cells remained below the BS surface, at a depth of 6–10 µm after 48 h (which appears in yellow).

#### 3.3.3. Scratch Assay

The effect of BS on HUVECs migration was studied through a scratch assay, following a previously reported procedure [27]. The migration of HUVECs cells was measured using image analysis software after 3, 6, 9, 12, and 24 h. 

**Figure 8:**
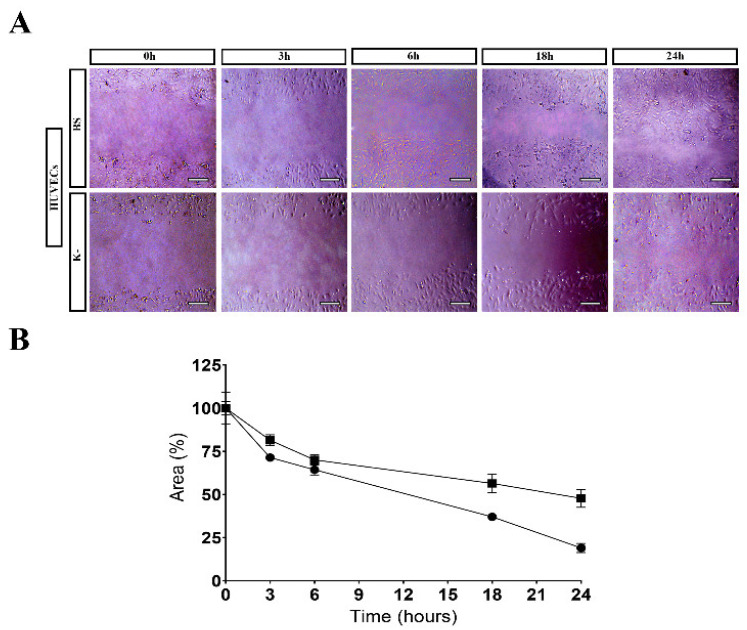
Results for scratch assay: (**A**) HUVECs migration in the presence of BS and control (K^−^); (**B**) Effect of the BS on the migration activity of HUVECs in the scratch assay. Data have been expressed as a percentage of cell area compared to the control. Scale bar: 100 µm.

Through the analysis of the results presented in Figure 8, it is possible to see that the BS induced the cell migration to the wound area, promoting an improved wound closure in comparison to the control group. Such results corroborated the ability of the products resulting from GG/KGM polysaccharide degradation (e.g., residues of glucose, mannose, glucuronic acid, and glucomannan) to encourage the cell migration [47,76,82]. 

Altogether, these results demonstrate the potential of GG/KGM as a bioink in the production of stable hydrogel matrices able to promote endothelial cell activity. In this study, the potential uses of the 3D printing techniques were also reinforced, being an emergent methodology that must be explored for the development of 3D living functional hydrogels that might surpass the current challenges present in the cardiac tissue regeneration field. For this paper, a simple and low-cost bioinks was developed to be used for the 3D printing of hydrogel structures for cardiac tissue regeneration. We intended to produce a natural material-based bioink that was suitable for 3D printing techniques without using any toxic solvents. This bioink conjugated two natural polymers (GG and KGM) with complementary characteristics. GG was chosen for structural proposes, as well as for its thermoresponsive characteristics, which would facilitate the 3D printing process. On the other hand, KGM was chosen to increase the bioactivity of the hydrogels. Consequently, these 3D structures presented a rough surface, which gave lots of anchorage points for cells, with an interconnected porous network. Moreover, the 3D printed hydrogels had suitable mechanical properties, with a controllable swelling and biodegradation profile. Finally, they exhibited biocompatible characteristics in contact with HUVEC, encouraging their adhesion to the BS’ surface, as well as encouraging internalization and migration; such events are crucial in the process of cardiac tissue formation. 

## 4. Conclusions

In this study, the suitability of natural polysaccharides-based bioink to produce 3D printed hydrogels was tested for the first time. To accomplish such a purpose, a combination of GG and KGM was prepared, and then the production of 3D printed hydrogel was successfully achieved. 

Our main findings were that the 3D printed structures presented mechanical, physicochemical, and biological properties adequate for cardiac tissue regeneration. Moreover, the 3D printed structures after the freeze-dried process remain stable, with similar mechanical integrity. Such properties will facilitate the upscaling of this technique, prompting the commercial interest of the produced structures. Overall, such pioneering work shows the potential of optimizing the natural compounds-based bioinks to produce 3D structures for biomedical applications, namely cardiac tissue restoration after myocardial infarction.

In the future, in vivo evaluation will be carried out, as well as bioink optimization for cell incorporation prior to the bioprinting process. The results presented will pave the way for the simple, low-cost, and efficient development of therapies for cardiac tissue regeneration using bioprinting.

## Data Availability

Data is available upon reasonable request.

## Supplementary Materials

The following supporting information can be downloaded at: https://www.mdpi.com/article/10.3390/polym15132824/s1, Supplementary Figure S1: 3D printing process of the KGM/GG scaffold; Supplementary Figure S2: ATR-FTIR spectra of KGM and GG pure materials compared with a KGM and GG blended hydrogel produced by casting, conventional scaffold (CS)—up, and by bioprinted scaffold (BS)—bottom.
