# Supplementary material for: Three-Dimensionally Printed Hydrogel Cardiac Patch for Infarct Regeneration Based on Natural Polysaccharides
Source: Polymers (Basel). 2023 Jun 26;15(13):2824. doi: 10.3390/polym15132824 (PMC10346776; doi:10.3390/polym15132824)
Supplement: Supplementary file 1 [file polymers-15-02824-s001.zip › polymers-2437168-supplementary.pdf]

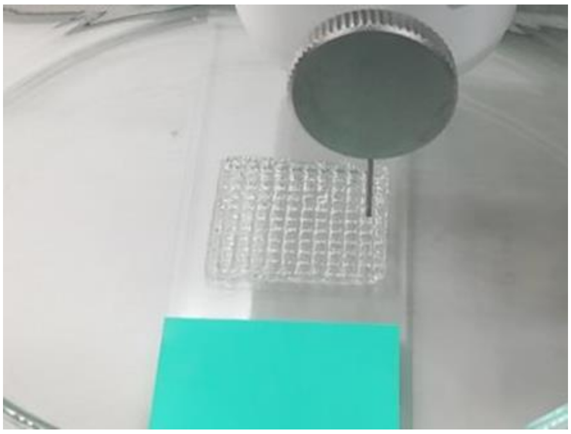

**Figure S1.** 3D printing process of the GG/KGM scaffold.

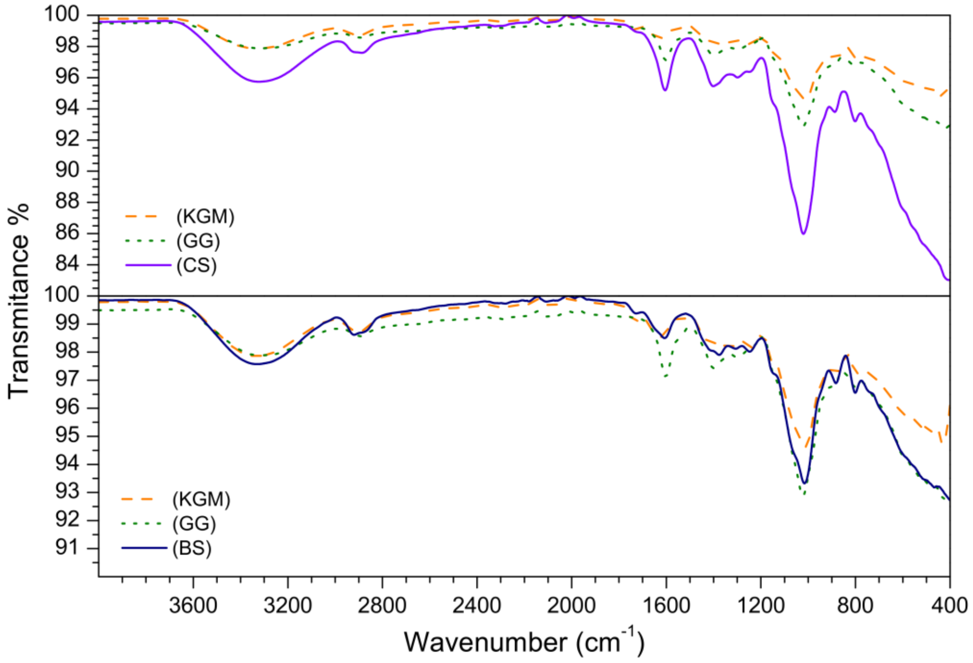


**Figure S2.** ATR-FTIR spectra of KGM and GG pure materials compared with a KGM and GG blended hydrogel produced by casting, conventional scaffold (CS)—up, and by bioprinted scaffold (BS)—bottom.
